# Withdrawn: Successful treatment of mucosal neuromas by radiofrequency ablation in a patient with multiple endocrine neoplasia type 2B

**DOI:** 10.1002/ski2.109

**Published:** 2022-07-28

## Abstract

Luis Escalante, Jennyfer Granizo‐Rubior, Esteban Ortiz‐Prado, Víctor Pinos‐León, Astrid Maldonado, David Chandler.^1^ Successful treatment of mucosal neuromas by radiofrequency ablation in a patient with multiple endocrine neoplasia type 2B. https://doi.org/10.1002/ski2.109

The above article from the Skin Health Disease published online on 28 July 2022 in Wiley Online Library (https://onlinelibrary.wiley.com/doi/10.1002/ski2.109) has been withdrawn by agreement among the authors, the Journal Editor‐in‐Chief George Millington and John Wiley & Sons Inc., Ltd on behalf of the British Association of Dermatology. The withdrawal has been agreed because the article was accepted and published online due to publisher error. This manuscript has been resubmitted and accepted as a Review Article and will publish with a new doi, https://doi.org/10.1002/ski2.146.
